# Biological parameters of *Rhipicephalus linnaei* determined using rabbits as experimental hosts under laboratory conditions

**DOI:** 10.1007/s10493-026-01175-2

**Published:** 2026-08-24

**Authors:** Joelly Santos, Marcos Valerio Garcia, Pâmella Oliveira Duarte, Leandra Marla Oshiro, Leandro de Higa, Álvaro Aragão, Jinávila Dandara de Rocha, Jacqueline Cavalcante Barros, Renato Andreotti

**Affiliations:** 1 Federal University of Mato Grosso do Sul (UFMS), MS Campo Grande, Brazil; 2 Tick Biology Laboratory, Embrapa Gado de Corte, MS Campo Grande, Brazil; 3 Federal University of Pelotas (UFPel), Rio Grande do Sul, RS Pelotas, Brazil

**Keywords:** Brown dog tick, Ixodidae, Molting, Oviposition, *Rhipicephalus sanguineus* sensu lato

## Abstract

*Rhipicephalus linnaei* is a tick widely associated with domestic dogs and is of recognized veterinary and public health importance. However, the recent taxonomic delimitation of this species, previously included within the *Rhipicephalus sanguineus* sensu lato complex, has made the production of species-specific biological data necessary. Therefore, this study aimed to characterize the life cycle parameters and reproductive performance of *R. linnaei* under controlled laboratory conditions. The specimens used were obtained from a laboratory colony established from engorged females collected from dogs, and artificial infestations were subsequently performed on rabbits. We evaluated the engorgement periods in larvae, nymphs, and adults; the molting period in the immature stage; and, in adult females, the body weight, preoviposition period, egg mass weight, incubation period, hatching rate, and nutritional conversion index. The results revealed significant differences among developmental stages, with an increase in the engorgement period from the larval stage to the adult stage and a longer molting period in the nymph stage. In adult females, a strong positive association was observed between body weight and egg mass weight, indicating a direct relationship between blood meal volume and reproductive investment. In addition, heavier egg masses were associated with higher hatching rates. These results provide updated information on the biology of *R. linnaei* and contribute to a more precise understanding of its dynamics under the currently accepted taxonomy.

## Introduction

Ticks of the family *Ixodidae* are hematophagous ectoparasites of major importance to One Health because they act as vectors of several pathogens affecting humans, domestic animals, and wildlife (Jongejan and Uilenberg 2004).

Among them, the group traditionally referred to as *Rhipicephalus sanguineus* sensu lato (s.l.) is notable for its close association with domestic dogs and its broad geographic distribution, especially in urban and peri-urban environments of tropical and subtropical regions (Dantas-Torres 2010; Šlapeta et al. 2021).

For decades, *R. sanguineus* s.l. was treated as a single cosmopolitan species. However, advances in morphological and molecular studies have demonstrated that this taxon corresponds to a complex of cryptic species with genetic, ecological, and biogeographic differences (Oliveira et al. 2005; Szabó et al. 2005; Burlini et al. 2009; Šlapeta et al. 2021, 2022). As a result of these revisions, *R. linnaei* (Audouin, 2021) was recognized as a valid and distinct species, corresponding to the so-called tropical lineage previously assigned to *R. sanguineus* s.l. (Šlapeta et al. 2022).

At present, *R. linnaei* is widely distributed in Brazil and occurs in different biomes, with records of coexistence between *Rhipicephalus sanguineus* sensu stricto and *R. linnaei* in some areas of southern Brazil (Caetano et al. 2017; Dantas-Torres et al. 2024). This broad distribution, together with its close association with anthropogenic environments and domestic hosts, reinforces the epidemiological relevance of the species. In this context, *R. linnaei* has been associated with several pathogens of veterinary and public health importance, including *Babesia canis*, *Coxiella burnetii*, *Ehrlichia canis*, *Rickettsia conorii*, and *Rickettsia rickettsii* (Dantas-Torres 2008). In addition, studies have expanded this scope by providing evidence for its potential role in the transmission of *Leishmania* spp. (Coutinho et al. 2005). The epidemiological relevance of hard ticks in Mato Grosso do Sul is further underscored by the detection of *Rickettsia* spp. in other ixodid species from the state (Almeida et al. 2013).

The species *R. linnaei* presents a set of characteristics that directly contribute to its ecological and epidemiological success. It is predominantly endophilic and adapted to remain and develop in indoor or peridomestic environments, especially those associated with domestic hosts. Its life cycle is three-host, in which each developmental stage (larva, nymph, and adult) requires a distinct host for blood feeding, and it is considered monotropic because all stages preferentially feed on the same host species, although it may adjust its survival strategies according to environmental conditions and host availability (Dantas-Torres 2010).

With respect to reproductive biology, *R. linnaei* has an oviposition period that may extend for several weeks, during which the total number of eggs laid by each female is directly related to body weight and to the duration of the oviposition period. This relationship reflects the reproductive energy investment of the species and represents an important biological parameter for understanding its population potential, especially under favorable environmental conditions (Koch 1982; Dantas-Torres 2010).

After oviposition, eggs pass through an interval of embryonic development of varied duration that may extend from a few days to several weeks depending on the environmental conditions to which they are exposed. Completion of this stage is marked by structural modifications in the egg envelope, when progressive rupture of the chorion can be observed along a characteristic longitudinal line, signaling the onset of larval emergence (Dantas-Torres 2008).

Despite these taxonomic and epidemiological advances, much of the available information on biological, reproductive, and developmental parameters attributed to *R. sanguineus* s.l. was obtained before the redefinition of the complex (Bechara et al. 1995), which hinders the precise attribution of such information to specific species such as *R. linnaei*.

In this context, the present study aimed to characterize in detail the main biological parameters of *R. linnaei* under controlled laboratory conditions, with emphasis on the duration of the different life stages, reproductive performance, and embryonic development. By generating species-specific data, this study seeks to fill gaps created by the historical attribution of biological information to the *R. sanguineus* s.l. complex and to contribute to a more accurate understanding of the biology of *R. linnaei*.

## Materials and methods

### Origin of the samples

The engorged females used to establish the colony were obtained locally from stray dogs at the Centro de Controle de Zoonoses (CCZ) of Campo Grande, Mato Grosso do Sul. Individual records of previous movement or place of origin were not available for these animals. Therefore, although the possibility that some dogs had previously come from other Brazilian regions cannot be completely excluded, this scenario was likely uncommon, given that the source population consisted of stray dogs captured locally by the CCZ. Initially, 20 engorged females were placed in a biochemical oxygen demand (BOD) incubator under controlled conditions for oviposition, egg incubation, and subsequent larval hatching. The first filial generation (F1) obtained from these females was subsequently used to establish a laboratory colony, which served as the source of specimens for the artificial infestations of rabbits (*Oryctolagus cuniculus*).

### Hosts and maintenance conditions

The rabbits used as experimental hosts were maintained in an institutional animal facility under standard laboratory conditions, with a nominal room temperature of 21 °C, a relative humidity between 50 and 70%, and a 12 h light:12 h dark photoperiod. All animal procedures followed the applicable ethical guidelines and were approved by the Committee on Ethics in the Use of Animals (CEUA) of Embrapa Gado de Corte under Protocol No. CEUA/001/2025.

### Tick maintenance in the laboratory

After natural detachment at each parasitic stage, the specimens were collected and transferred to a climate chamber (SL-206 Solab), which was maintained at 27 ± 1 °C, a relative humidity of 80–90%, and a 12 h light:12 h dark photoperiod. Under these conditions, the specimens were kept individually and monitored daily for postparasitic development. In adult females, oviposition, egg incubation, and hatching were monitored under the same controlled conditions, and engorged female weight was recorded before the onset of oviposition using an analytical balance.

### Biological parameters evaluated

Biological parameters were evaluated throughout the life cycle of *R. linnaei*, including the engorgement period in larvae, nymphs, and adults; the molting period in the immature stages; and, in adult females, the engorged female weight, preoviposition period, egg mass weight, incubation period, hatching rate, and nutritional conversion index (NCI). The engorgement period was defined as the number of days from infestation to natural detachment, and the molting period was defined as the interval between the detachment of engorged immature stages and ecdysis. In adult females, the preoviposition period was defined as the interval between natural detachment and the onset of oviposition, whereas the incubation period was defined as the interval between the onset of oviposition and larval hatching. The hatching rate was calculated as the percentage of eggs that successfully hatched under controlled laboratory conditions, and the NCI was calculated as the ratio between egg mass weight and engorged female weight, expressed as a percentage. Oviposition, incubation, and hatching were monitored by daily observation.

### Statistical analyses

Statistical analyses were performed in the R environment (R Core Team 2024), with data organization and standardization conducted using packages from the tidyverse ecosystem (Wickham et al. 2019). Descriptive statistics were calculated separately for each developmental stage, considering only valid observations for each variable.

Because the data were asymmetric, the sample sizes were unequal, and biologically plausible extreme values were present; thus, nonparametric methods were adopted. Comparisons among developmental stages were performed using the Kruskal‒Wallis test, followed by Dunn’s post hoc test with Benjamini‒Hochberg correction when applicable. For variables evaluated only in the immature stages, the Wilcoxon test was used.

Relationships among reproductive parameters were evaluated exclusively in adult females using Spearman correlation. In addition, a simple linear regression was fitted between engorged female weight and egg mass weight for descriptive purposes.

## Results

The life cycle of *R. linnaei* maintained under laboratory conditions showed consistent variation among developmental stages, reflecting physiological and functional differences throughout the cycle. The evaluated parameters were analyzed separately by stage (Tables 1 and 2), considering only valid observations for each variable.

**Table 1:** Biological parameters of *R. linnaei* larvae and nymphs maintained under laboratory conditions. Values are presented as means ± standard deviations, with the number of valid observations given in parentheses

Parameter	Larvae	Nymphs
Engorgement period (days)	5.71 ± 1.33 (24)	8.62 ± 4.00 (29)
Molting period (days)	11.87 ± 0.76 (23)	17.86 ± 1.58 (22)
Molting rate (%)	97.21 ± 4.98 (24)	97.50 ± 6.12 (6)
Individual weight (mg)	0.44 ± 0.72 (23)	2.98 ± 0.57 (29)

**Table 2:** Biological and reproductive parameters of adult *R. linnaei* females maintained under laboratory conditions. Values are expressed as means ± standard deviations; the number of valid observations is given in parentheses

Parameter	Value
Engorgement period (days)	10.85 ± 1.56 (59)
Engorged female weight (mg)	148.45 ± 41.57 (59)
Preoviposition period (days)	3.34 ± 0.69 (58)
Egg mass weight (mg)	87.03 ± 34.00 (59)
Incubation period (days)	30.02 ± 4.71 (44)
Hatching rate (%)	93.50 ± 20.18 (59)
Nutritional conversion index (%)	58.23 ± 11.16 (58)

**Fig. 1:**
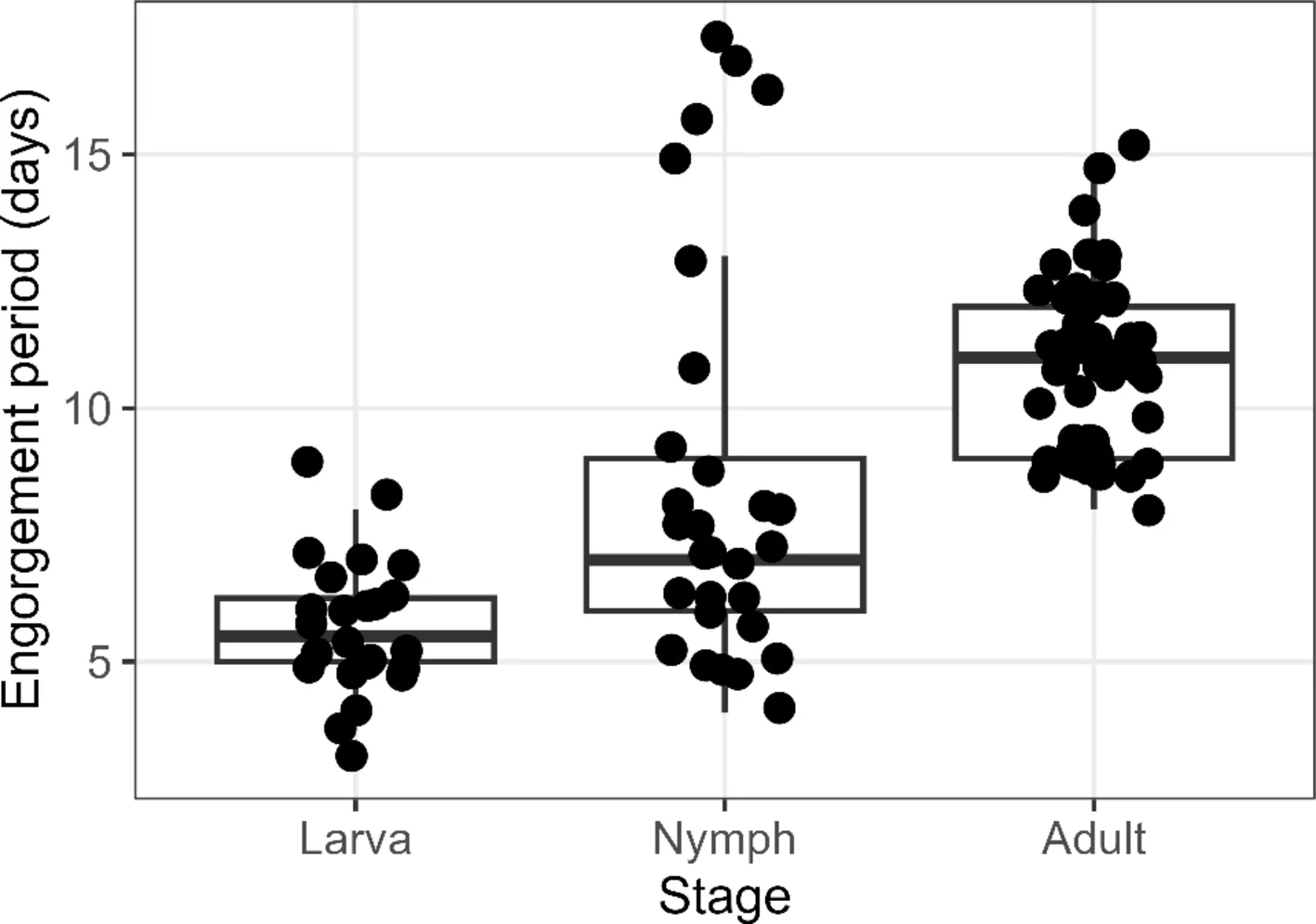
Distribution of the engorgement period (days) across developmental stages (larva, nymph, and adult). Boxes represent the interquartile range (IQR), the central line indicates the median, and points correspond to individual observations

The engorgement period differed significantly among the larvae, nymphs, and adults (Kruskal‒Wallis, *p* < 0.001). Larvae presented the lowest values, followed by nymphs, whereas adults exhibited the longest engorgement periods (Fig. 1). Dunn’s post hoc test indicated significant differences among all pairs of stages (larva × nymph, adjusted *p* = 0.0027; larva × adult, adjusted *p* < 0.001; nymph × adult, adjusted *p* < 0.001).

**Fig. 2:**
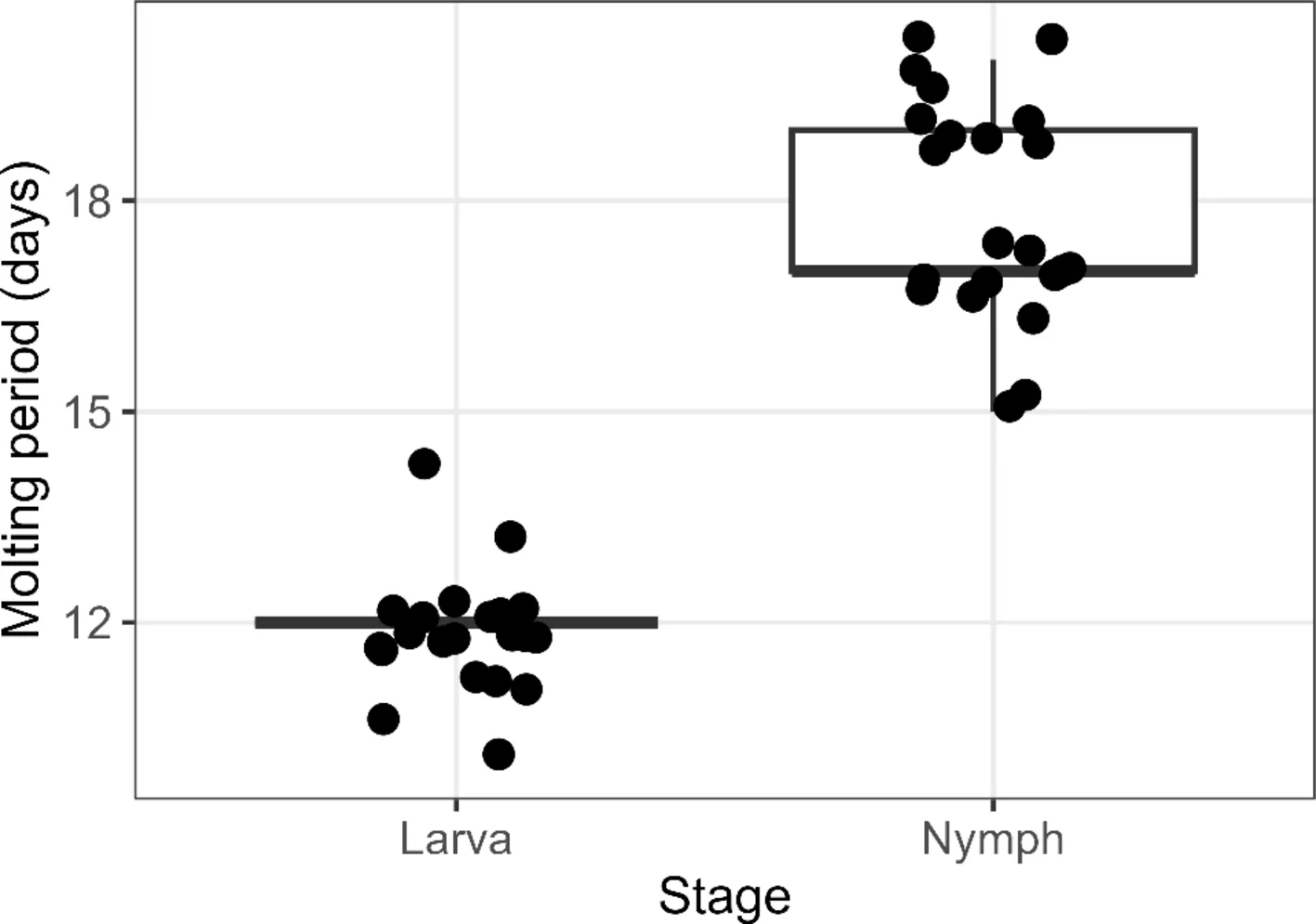
Molting period (days) in the larval and nymphal stages. Boxes represent the interquartile range (IQR), the central line indicates the median, and points correspond to individual observations

The molting period, recorded only for the immature stages, differed significantly between the larvae and nymphs (Wilcoxon test, *p* < 0.001). Compared with larvae, nymphs exhibited consistently longer molting periods, indicating a greater time requirement for morphophysiological reorganization at this developmental stage (Fig. 2).

**Fig. 3:**
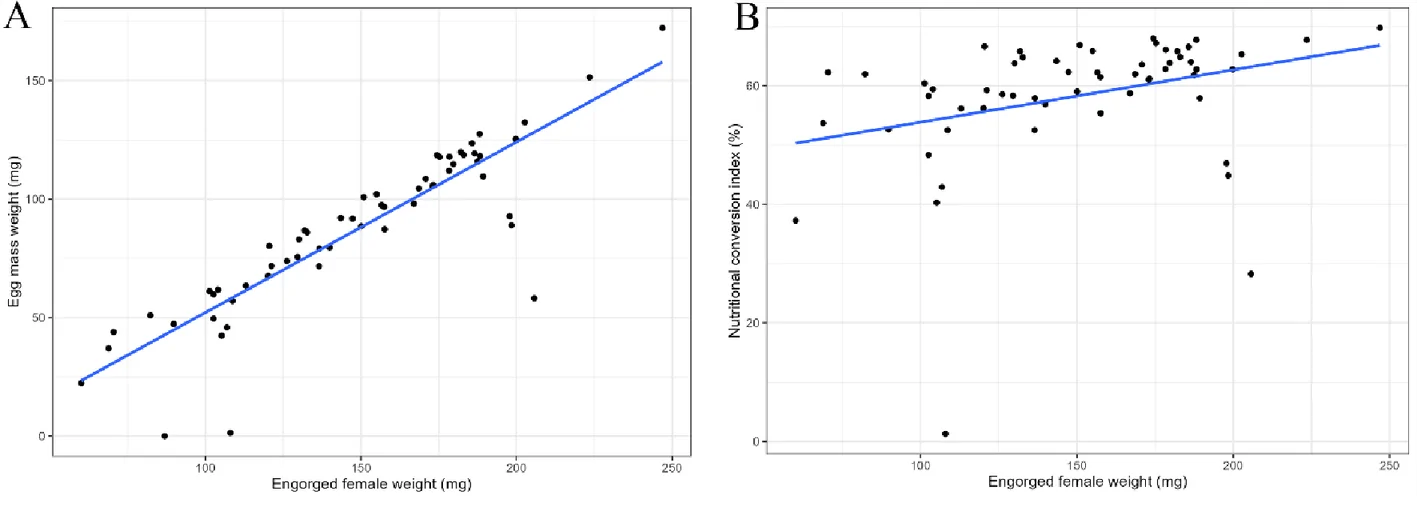
Relationships between engorged female weight and reproductive parameters in adult females. (**A**) Relationship between engorged female weight (mg) and egg mass weight (mg). (**B**) Relationship between engorged female weight (mg) and nutritional conversion index (NCI, %). Points represent individual observations, and lines indicate the linear fit used to visualize the general pattern

Reproductive analyses were conducted exclusively with adult females. A strong positive correlation was observed between engorged female weight and egg mass weight (Spearman, rho = 0.878, *p* < 0.001), indicating that females with greater body mass produced heavier egg masses (Fig. 3a). This relationship was corroborated by simple linear regression, which revealed a significant positive effect of female weight on egg mass weight (beta = 0.720; *p* < 0.001; R2 = 0.775), demonstrating that a substantial portion of the variation in egg production was explained by female body mass.

**Fig. 4:**
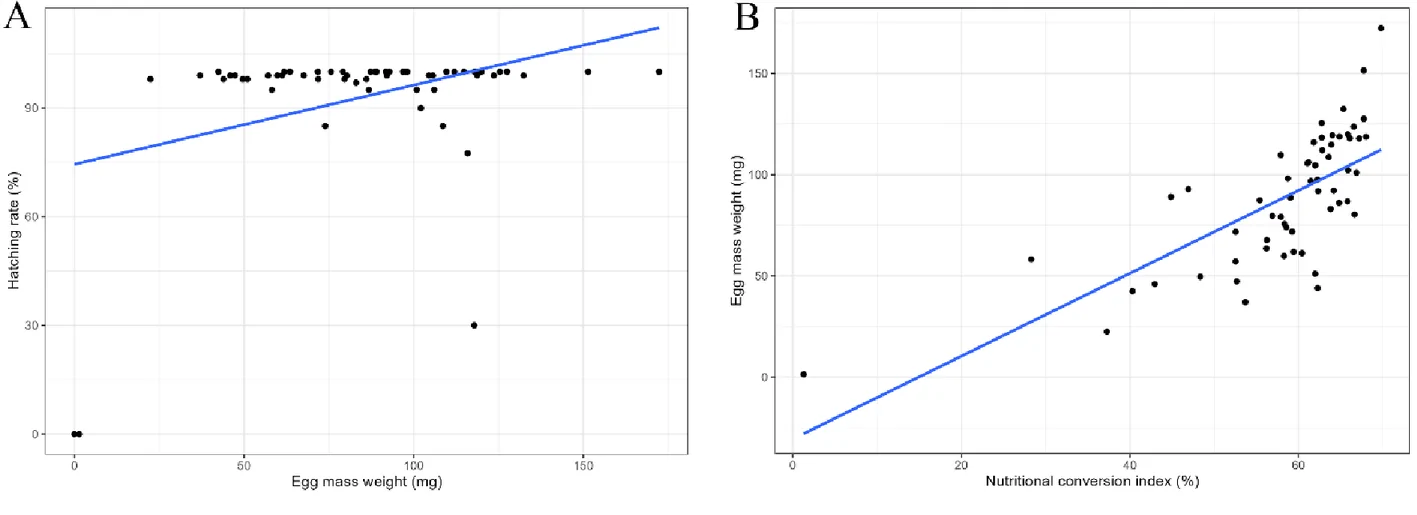
Relationships among reproductive parameters in adult females. (**A**) Relationship between egg mass weight (mg) and hatching rate (%). (**B**) Relationship between nutritional conversion index (NCI, %) and egg mass weight (mg). Points represent individual observations, and lines indicate the general trend of the data

The NCI was moderately positively correlated with engorged female weight (Spearman, rho = 0.445; *p* < 0.001), suggesting a positive correlation between female body mass and the conversion of the blood meal into reproductive biomass (Fig. 3b). In addition, a significant positive correlation was observed between egg mass weight and hatching rate (Spearman, rho = 0.338, *p* = 0.0088), indicating that heavier egg masses tended to show higher hatching rates under the laboratory conditions evaluated (Fig. 4a). Similarly, the NCI was significantly positively correlated with egg mass weight (Spearman, rho = 0.752; *p* < 0.001; Fig. 4b).

## Discussion

Taken together, the results show that the life cycle parameters of *R. linnaei* vary significantly among developmental stages and that, in adult females, egg production is strongly associated with body mass. In addition, egg mass weight was positively associated with the hatching rate, indicating that greater reproductive investment did not imply a reduction in embryonic viability under the laboratory conditions evaluated.

The immature-stage parameters observed here were broadly consistent with those previously reported by Bechara et al. (1995) under laboratory conditions. Larval engorgement and molting periods were very similar between studies, indicating close agreement in the timing of larval development. In nymphs, body weight was also comparable, although the engorgement period was slightly longer and the molting period shorter in the present study. Overall, these comparisons indicate that our data largely corroborate earlier laboratory observations for immature stages and suggest that some aspects of nymphal development may vary according to host use, colony maintenance, or environmental conditions.

The difference among stages in terms of feeding duration observed here is consistent with the biology of ixodid ticks and, more specifically, with the pattern described for the *Rhipicephalus sanguineus* sensu lato complex, in which feeding duration varies according to developmental stage and host (Dantas-Torres 2010). Classical studies on tick biology have already shown that larvae complete feeding in a short time, nymphs tend to prolong this period, and adult females may remain attached for longer intervals before detachment, a pattern that was also observed in the present study (Nuttall 1915; Pegram et al. 1987). However, direct comparisons require caution because life cycle parameters, including infestation performance and the duration of off-host stages, may vary across hosts, environmental conditions, and species or lineages within the complex, in addition to differences in colony maintenance (Dantas-Torres 2010; Labruna et al. 2017; Tian et al. 2022).

The significantly longer molting period in nymphs than in larvae is biologically plausible because the nymphal molt likely requires more time due to the processes of remodeling, formation, and hardening of the new cuticle. In addition, recent studies have indicated that development, including molting time, is strongly dependent on temperature and, to a lesser extent, relative humidity, with shorter developmental times occurring at higher temperatures within permissive ranges (Leal et al. 2020; Tian et al. 2022). This may help explain why, although our results are generally comparable to those of Bechara et al. (1995), some differences were observed, especially in the nymphal stage.

For adult females, the present findings both corroborate and refine those reported by Bechara et al. (1995). The engorgement period recorded here was longer than the values previously described for ticks fed on dogs, foxes, guinea pigs, and hamsters. Similarly, the mean engorged female weight and egg mass weight were relatively high, matching or surpassing the highest values reported for the host groups examined in that study. Together, these results suggest that adult females in our experimental system attained high engorgement levels and substantial reproductive output. In contrast, the preoviposition period was short and remained within the range previously reported, showing the greatest similarity to the values observed for hamsters.

The reproductive component of the present study further reinforces the central role of engorgement in the reproductive success of the species. The strong association between engorged female weight and egg mass weight indicates that variation in oviposition is largely explained by the amount of resources obtained during feeding, as much of the nutritional resources allocated to vitellogenesis are derived directly from the blood ingested during feeding (Uspensky and Ioffe-Uspensky 1999; Gaxiola-Camacho et al. 2009). Studies on the reproductive biology of ixodid ticks have also demonstrated that female body weight after engorgement is associated with oviposition output (Campbell and Glines 1979; Drummond et al. 1971). Although Bechara et al. (1995) did not test this relationship statistically, their results also indicate that host groups with heavier engorged females tended to produce heavier egg masses. In this sense, the results of the present study not only corroborate this general reproductive pattern but also quantify it statistically in *R. linnaei*.

The incubation period, in turn, was longer in the present study than in that of Bechara et al. (1995), whose values were generally shorter across host groups. This difference may be related, at least in part, to differences in colony maintenance, host use, or laboratory conditions, particularly temperature, as embryonic development in ticks is sensitive to environmental variation. Even so, the hatching rate in the present study remained high and within the upper range of values previously reported under laboratory conditions. Moreover, the positive correlation between egg mass weight and hatching rate suggests that, under the conditions evaluated here, heavier egg masses did not imply a proportional loss of embryonic viability.

The NCI was positively associated with engorged female weight, indicating that females with greater blood intake were more efficient at converting this resource into reproductive biomass. Although this index has rarely been explicitly examined in the literature, it is well established that the blood meal constitutes the primary nutritional source for vitellogenesis and ovarian development in ixodid ticks. Because Bechara et al. (1995) did not report this parameter, a direct comparison is not possible. Therefore, the evaluation of nutritional conversion efficiency represents an additional contribution of the present study, complementing the traditional reproductive parameters usually reported for this group.

The results reported here were obtained under controlled laboratory conditions and with an experimental host, which may influence feeding, molting, and reproductive parameters in comparison with what occurs in dogs and under natural conditions. This is particularly relevant when the present data are compared with those of Bechara et al. (1995), who evaluated adult females on different host species and immature stages of tick-bite–naive guinea pigs. In addition, although the design allowed estimation of the main life cycle parameters, some outcomes were calculated using different sample sizes across stages, which recommends caution in the extrapolation of specific relationships. Thus, the values reported here should be interpreted as a standardized reference for the species that is useful for comparisons but that may vary under different environmental regimes, hosts, and populations.

Much of the information available on the biological parameters of ticks of the genus *Rhipicephalus* was produced before the taxonomic revisions that redefined and redescribed species formerly treated under a broad concept. As a consequence, many classical data on life cycle, reproduction, and physiological performance were originally attributed to taxonomic complexes that are now recognized as distinct, which restricts their direct application to the currently delimited species. In this sense, the present study both corroborates and refines previously published laboratory data. Several biological patterns reported here are broadly consistent with those described by Bechara et al. (1995), especially for immature development and hatching success. Moreover, differences were observed in the adult engorgement period, female weight, egg mass weight, and incubation period. Therefore, the contribution of the present study is not only a repetition of earlier observations but also a reinterpretation and update these biological parameters specifically for *R. linnaei* under the currently accepted taxonomy.

## Data Availability

We declare that all data are provided within this manuscript.
